# Estimation of Chemical Composition of Pork Trimmings by Use of Density Measurement—Hydrostatic Method

**DOI:** 10.3390/molecules25071736

**Published:** 2020-04-09

**Authors:** Lech Adamczak, Marta Chmiel, Tomasz Florowski, Dorota Pietrzak

**Affiliations:** Division of Meat Technology, Department of Food Technology, Faculty of Food Sciences, Warsaw University of Life Sciences-SGGW, 02-787 Warsaw, Poland; marta_chmiel@sggw.pl (M.C.); tomasz_florowski@sggw.pl (T.F.); Dorota_pietrzak@sggw.pl (D.P.)

**Keywords:** density, hydrostatic method, chemical composition, pork, quality

## Abstract

This study aims to determine the possibility of using density measurements by using the hydrostatic method for the estimation of the chemical composition of pork. The research material included 75 pork samples obtained during industrial butchering and cutting. The density measurements were performed using the hydrostatic method based on Archimedes’ principle. The meat samples were minced, and the content of the basic chemical components in them was determined. The usefulness of density measurement using the hydrostatic method in chemical composition estimation was determined by analyzing the correlation for the entire population, and after grouping the samples with a low (<15%), medium (15–25%), and high (>25%) fat content. High (in absolute value) coefficients of correlation between the meat density and the content of water (0.96), protein (0.94), and fat (−0.96) were found based on the results obtained. In order to achieve higher accuracy of the estimation, the applied regression equations should be adjusted to the presumed fat content in the meat. The standard error of prediction (SEP) values ranged from 0.67% to 2.82%, which indicates that the calculated estimation accuracy may be sufficient for proper planning of the production. Higher SEP values were found in fat content estimation and the lowest ones were found in protein content estimation.

## 1. Introduction

The quality of meat products is closely linked to the quality of the raw materials used. One of the basic parameters of meat and meat products’ quality is their chemical composition. The content of water, fat, and protein affects both the technological and processing quality of the meat, as well as the quality of the finished product. Knowledge of these components in the raw material is important to ensure reproducible production [1,2,3,4,5]. This also has legal significance, resulting from mandatory nutritional labeling in the European Union [6]. The standardization of raw materials can also affect the efficiency of production due to differences in the price of meat with different fat contents. In the case of pork meat used for cold cuts production, the highest differentiation is observed in the fat content [7,8]. Based on the fat content, meat trimmings are classified into different classes (e.g., lean, medium-fat, and fat). Conventional basic chemical composition tests of meat rely on chemical analyses, which are laborious, time-consuming, complicated, costly, destructive, and sometimes harmful to the environment due to the usage of different chemical reagents [9]. For this reason, they are used only to examine small samples collected from a larger batch of products. This, in turn, cannot ensure their representativeness.

The search for new, non-invasive, quick, and low-cost methods for the analysis of the chemical composition of meat and other raw materials is the subject of many studies [10]. For example, there were some attempts to use near infra-red spectrometry (NIRS) [9,11], dual energy x-ray (DXR) [4,12,13], ultrasound [14,15], computed tomography (CT) [16], and 3D scanning [17,18] for this purpose. Among the methods for density determination, the hydrostatic method (using Archimedes’ principle) is the simplest to use, and also the cheapest. The first attempts at using density measurements by the hydrostatic method to determine the fatness of pork half-carcasses were already made in the 1950s [19]. The control of the chemical composition of meat is important for the quality of meat products and for correctly informing consumers about the nutritional value of products. The possible introduction of the hydrostatic method into industrial practice, for determining the chemical composition of meat, does not require large financial expenditures. The simplicity of the method also does not require the specialized training of employees. Its use will allow for the optimal management of raw materials and reduce the number of samples subjected to traditional control using the reference methods.

Therefore, an attempt to use the hydrostatic method for the density measurements in the estimation of pork chemical composition was made in this study.

## 2. Results and Discussion

### 2.1. Meat Density and Its Chemical Composition—Theoretical Considerations

The density of meat can be theoretically correlated with the fat content using the principle of additivity/sum of fractions/“Feder number”:

1) Principle of density additivity:
  ρ = (ρ_w_x_w_ + ρ_p_x_p_ + ρ_f_x_f_ + ρ_a_x_a_) (1)
  where x_w_, x_p_, x_f_, x_a_—weight fractions of water, protein, and fat, and ash, respectively
  ρ_w,_ ρ_p_, ρ_f_, ρ_a_—density of water, protein, fat, and ash, respectively,
2) Sum of mass fractions of the components equal to the unity:
  1 = x_w_ + x_p_ + x_f_ + x_a_ (2)
3) “Feder number” [20,21,22], for determining the ratio of water (x_w_) to protein (x_p_) content:

N_Fe_ = x_w_/x_p_ (3)

Based on formulas 1, 2, and 3, and assuming that the ash content in the meat is at a level of 1.0%, the following relationship is obtained after the transformations:

ρ = x_f_ × (ρ_f_ × (N_Fe_ + 1) − ρ_w_ N_Fe_ − ρ_p_)/(N_Fe_ + 1) + (0.99 × ρ_w_ × N_Fe_ + 0.99 × ρ_p_)/(N_Fe_ + 1) + 0.01 × ρ_a_ (4)

The expanded transformation of the formula was reported in Adamczak et al. [17]

Assuming the slight variability of N_Fe_, and assuming that fat density is lower than the density of water and protein, it can be concluded that system density is proportional to the content of fat (**x_f_**). Therefore, there is a theoretical possibility of correlating the density with fat content and, consequently, with the content of water and protein.

Relationships between water and protein content based on “Feder number” are used in the EU procedures for the determination of meat content in processed pork. They are also used to determine the amount of water absorbed by the chicken carcasses during post-slaughter cooling [23,24].

### 2.2. Characteristics of Research Samples

The examined samples of pork meat were divided into three groups depending on their fat content (Table 1). Samples with a higher fat content and a lower content of protein and water were of lower density. The average water content for the entire examined population was 60.5%, protein 17.5%, and fat 21.9% (Table 1). The fat content, in accordance with the assumptions, varied in a wide range from 5.0% to 46.9%, with a density of 1.0052 to 1.0622 g cm^−3^. The density of meat obtained from pork ham was at a slightly higher level of 1.0823–1.0997 g cm^−3^ in the study conducted by Sosa-Morales et al. [25] The variation in the results could be due to the different method of density measurement used in the studies of the above-mentioned authors (based on external dimensions and weight) and the use of lean meat in their study.

It should be noted that the use of the hydrostatic method for density determination may be restricted by the high fat content in the meat. The samples characterized by fat content of approximately 50% have a density similar to the density of water, so there is no possibility of determining their weight after immersion.

The ratio of average water content to protein (Feder number, x_w_/x_p_) in the lean meat (fat content below 15%) was 3.43. In turn, in medium-fat (15–25%) and fat meat (above 25%), there was a slight change in this ratio, which was 3.47 and 3.52, respectively. This indicates the correctness of the theoretical assumptions adopted in this study (slight N_Fe_ variability).

### 2.3. Density Measured by Hydrostatic Method and Chemical Composition of Pork

The relationship between the density measured by the hydrostatic method and the content of basic chemical components in the meat was determined based on the correlation analysis. The obtained correlation coefficients and regression equations are summarized in Table 2. The significance (*p* < 0.05) of all the obtained correlation coefficients was found, regardless of the analyzed meat group, for the entire population of samples. The highest (in absolute value) correlation coefficients were found between the density and fat content in all the analyzed meat groups. Additionally, the presence of higher (in absolute value) correlation coefficients between meat chemical components and their density was noted in the meat containing more than 25% fat, compared to the leaner meat (Table 2).

**Figure 1:**
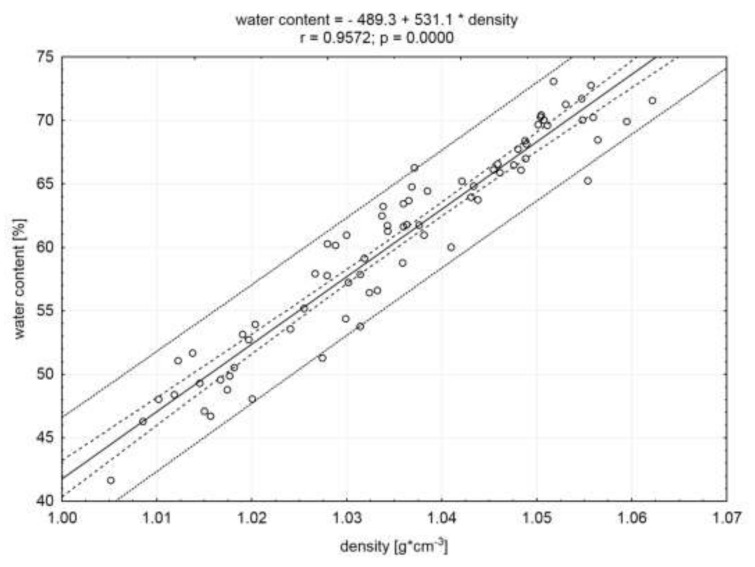
Correlation between water content and density of the meat. Dotted lines indicate the confidence and prediction curves (for the level of 0.95).

**Figure 2:**
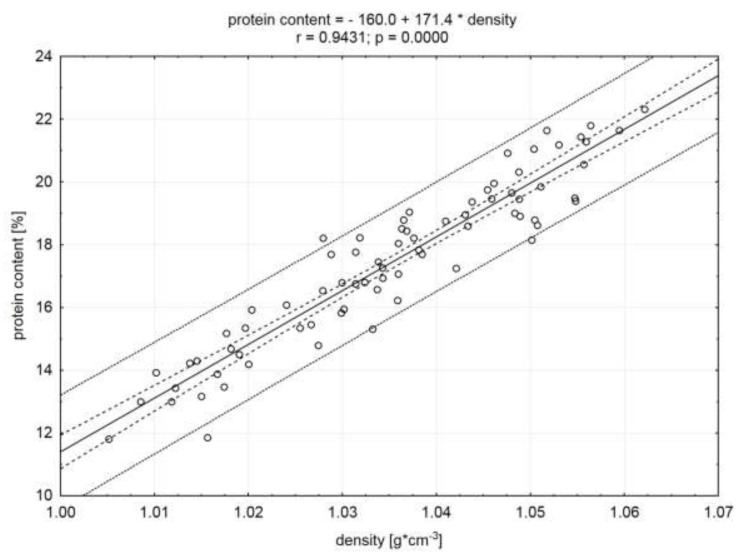
Correlation between protein content and density of the meat. Dotted lines indicate the confidence and prediction curves (for the level of 0.95).

**Figure 3:**
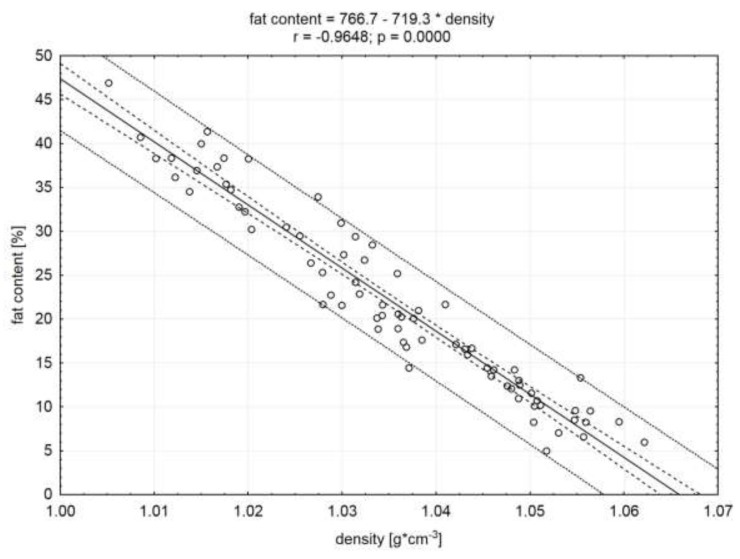
Correlation between fat content and density of the meat. Dotted lines indicate the confidence and prediction curves (for the level of 0.95).

The significant (*p* < 0.05) and the highest (in absolute value) correlation coefficients were demonstrated, based on the statistical analysis conducted for the whole population of examined raw material (n = 75) (Table 2). The obtained relationships are shown in Figure 1, Figure 2, and Figure 3. The calculated correlation coefficients between protein and water content and density were r = 0.94 and r = 0.96, respectively (Figure 1 and Figure 2). Additionally, a high (in absolute value) correlation coefficient was found between the fat content and meat density (r = −0.96). A negative value of this coefficient indicates an inverse proportional relationship (Figure 3). Adamczak et al. [17], in the study using 3D scanning, in order to determine the density of pork necks and to correlate it with basic chemical compounds’ content, obtained significant correlation coefficients. However, their values (absolute) were lower and did not exceed 0.66 (correlation between the density and fat content).

The considerable variability of regression equations (values of coefficients of equations), in particular meat groups, means that while attempting to use the hydrostatic method of density determination for meat chemical composition estimation, different regression equations should be used individually, depending on the scope of fat content in the meat.

The values of standard error of prediction (SEP) indicate that the accuracy of the estimation of the content of water, protein, and fat, based on the density determined using the hydrostatic method of the examined meat, oscillated between 0.67% (for the estimation of protein content in the group containing 15–25% fat) and 2.82% (for the estimation of fat content in the whole examined population). Higher SEP values were found in the fat content estimation, and the lowest ones in the protein estimation (Table 2). It seems that the calculated estimation accuracy may be sufficient for proper planning of the production process and possible listing of the nutritional information on the label. According to Nowak et al. [15], the use of density measurements using ultrasounds for an estimation of meat products’ chemical composition allows one to obtain average deviations in the content of water, protein, and fat at the level of 2.3%, 0.8%, and 1.8%, respectively. By using this combined method (combination of ultrasonic method and density measurement), higher deviations in the estimation with respect to the values determined using reference methods were noted for products containing more than 20% fat. During the method validation, higher accuracy than forecasted on the basis of SEP was obtained, regardless of the analyzed group and for the whole sample population examined (Table 2). This proves the correctness of the inference based on the calculated values of the prediction coefficients. The method is accurate and adequate for application in real samples.

Taking into account the ratio of SEP to average values of the entire examined population, for protein content it amounts to 5.0% and for fat it is 13.1%. According to the EU regulations [26], the declaration of protein or fat of the content on the label is in the range of 10–40%, and the permissible tolerance limit is 20% of the declared value. Based on the results obtained in this study, it can be concluded that the accuracy of estimation is sufficient for the purposes of the declaration of products’ nutritional value on the labels. As per the final products, the yield in the production process should also be taken into account.

## 3. Materials and Methods

### 3.1. Study Samples

The study materials were samples of pork, with varying fat contents. The mass of individual pieces approximated 200 g. The samples were collected during the cutting and trimming processes in industrial conditions. Meat was obtained during the cutting and trimming of ham and shoulder muscles. Sample selection was performed directly on the line. A total of 75 pork samples were collected for the study.

The selection of raw material from industrial production allows one to minimalize the possible influence of intravital factors on the accuracy of estimating the chemical composition of meat.

### 3.2. Methods

#### 3.2.1. Density Measurement Using Hydrostatic Method

The density measurement using the hydrostatic method is based on Archimedes’ principle. The AZG 4000C scales (Axis, Gdańsk, Poland) with a precision of 0.01 g were used for density measurements. Each of the 75 pork samples were weighed first in air (sample temperature 4 °C, air temperature 18–20 °C) and then after immersion in distilled water at a known temperature (4–5 °C). The density of meat samples was calculated according to the following formula (5):

ρ = A/(A − B) × ρ_0_ (5)

where:

ρ-sample density at measurement temperature (g cm^−3^),
A—sample weight in air (g),
B—sample weight in liquid (g),
ρ_0_-liquid density with respect to temperature measurement (g cm^−3^).

After measurements, the samples were dried using filtering paper. These samples were then used to determine the basic chemical composition of the meat.

Each of the meat samples were ground in the grinder (MESKO, Skarżysko Kamienna, Poland) with a 2 mm diameter hole, and were homogenized by mixing. The content of water, fat, and protein in the samples was determined using the appellate methods [27]. The moisture content was determined by drying samples at 105 °C (SUP-65 dryer, Wamed, Warsaw, Poland). The protein content was determined by the Kjeldahl method (Velp Scientifica UDK 129 Distillation Unit, Usmate, Italy). The fat content was determined by Soxhlet extraction (Büchi Extraction System B-811, Büchi, Warsaw, Poland).

#### 3.2.2. Method Validation—Accuracy

The accuracy of the method was verified by comparing the results obtained with the validated method (estimation based on density), with the results obtained with the reference methods. Nine randomly selected meat samples for each of the analyzed groups were tested (meat was obtained as described in Section 3.1.). Therefore, 27 meat samples were used to determine the accuracy of the estimation based on the regression equation for the entire population.

The accuracy of the method was calculated according to formula (6) as the absolute error of each measurement.

Re = |x – y|(6)

where:

Re—absolute error [%],
x—value estimated based on the regression equation,
y—value determined by reference methods.

The obtained value of the absolute error, for 9 measurements done in each group and 27 for the whole population, was averaged and presented as the mean absolute error.

#### 3.2.3. Statistical Analysis

The samples were divided into three groups based on the results obtained using the reference methods of fat determination. The division criterion was fat content: up to 15% (n = 25), 15–25% (n = 22), and above 25% (n = 28). A similar division (based on the fat content) into lean, medium-fat, and fat meat is carried out by employees of cutting lines when cutting meat trimmings. The results were subject to statistical analysis using Statistica ver. 12 PL (StatSoft Inc., Tulsa, OK., USA). The relationships between the density of meat determined by the hydrostatic method and the content of individual chemical components were determined using the Pearson’s (linear) correlation. The linear relationship results from theoretical assumptions (see Equation (4)). The correlation coefficients and the regression equations between the content of water, protein, and fat and the density of examined meat were presented. The standard error of prediction (SEP) was calculated for the purpose of determination of the accuracy of individual chemical compounds’ estimation based on meat density.

## 4. Conclusions

Determination of density by the hydrostatic method could be used to estimate the water, protein and fat content in pork trimmings. This is indicated by high (in absolute value) coefficients of correlation between the above-mentioned properties obtained in the study. Depending on the range of fat content in meat, differentiated regression equations were obtained, but the highest correlation coefficients were found for the whole population of examined raw material. The standard error of prediction (SEP) for the entire examined sample population did not exceed 20% of the average content of protein and fat, which is the sufficient accuracy for the declaration of nutritional value on a product’s label. The hydrostatic method for the estimation of meat chemical composition has its limitation which is the maximum fat content of about 50%. This limitation is caused by the similar density of meat and water in that situation, and by the inability to determine meat weight after immersion. Due to the accuracy of the method, it can be used for a general estimation of the content of chemical components in meat, but it is not an analytical method.

## Figures and Tables

**Table 1:** Density, basic chemical composition and water sorption of the analyzed pork meat.

Meat Group	Discriminant	Average	Minimum	Maximum	SD
fat content 15% n = 25	density [g cm^−3^]	1.0511	1.0371	1.0622	0.0052
	water content [%]	68.9	65.3	73.1	2.3
	protein content [%]	20.1	18.1	22.3	1.2
	fat content [%]	10.6	5.0	14.4	2.8
fat content 15–25% n = 22	density [g cm^−3^]	1.0361	1.0280	1.0438	0.0046
	water content [%]	62.2	57.9	65.2	2.0
	protein content [%]	17.9	16.6	19.4	0.8
	fat content [%]	19.8	15.9	24.2	2.4
fat content 25% n = 28	density [g cm^−3^]	1.0207	1.0052	1.0359	0.0082
	water content [%]	51.8	41.6	58.8	4.2
	protein content [%]	14.7	11.8	16.8	1.4
	fat content [%]	33.8	25.2	46.9	5.5
total n = 75	density [g cm^−3^]	1.0354	1.0052	1.0622	0.0143
	water content [%]	60.5	41.6	73.1	7.9
	protein content [%]	17.5	11.8	22.3	2.6
	fat content [%]	21.9	5.0	46.9	10.7

**Table 2:** Correlation coefficients, regression equations and SEP (Standard Error of Prediction) between density determined by hydrostatic method and basic chemical composition of the analyzed pork meat, method accuracy.

Characteristics	Density of Particular Meat Groups			
	Fat Content 15% n = 25	Fat Content 15–25% n = 22	Fat Content 25% n = 28	Total n = 75
r				
Water	0.60 *	0.65 *	0.87 *	0.96 *
Protein	0.60 *	0.61 *	0.84 *	0.94 *
Fat	−0.73 *	−0.75 *	−0.88 *	−0.96 *
regression equation				
Water	y = −210.1 + 265.5 x	y = −229.3 + 281.3 x	y = −403.2 + 445.8 x	y = −489.3 + 531.1 x
Protein	y = −122.1 + 135.3 x	y = −67.9 + 82.8 x	y = −130.7 + 142.5 x	y = −160.0 + 171.4 x
Fat	y = 428.8 − 397.9 x	y = 415.3 − 381.8 x	y = 638.2 − 592.1 x	y = 766.7 − 719.3 x
SEP				
Water	1.88	1.55	2.11	2.31
Protein	0.95	0.67	0.77	0.87
Fat	1.99	1.60	2.66	2.82
Validation - accuracy	n = 9	n = 9	n = 9	n = 27
Water	1.21	1.09	1.73	1.34
Protein	0.72	0.61	0.51	0.62
Fat	0.49	0.48	0.97	0.65

* significant coefficient at *p* < 0.05.

## References

[1] Brienne J.P., Denoyelle C., Baussart H., Daudin J.D. (2001). Assessment of meat fat content using dual energy X-ray absorption. Meat Sci..

[2] Campagnol P.C.B., dos Santos B.A., Wagner R., Terra N.N., Pollonio M.A.R. (2012). Amorphous cellulose gel as a fat substitute in fermented sausages. Meat Sci..

[3] Ham Y.K., Hwang K.O., Kim H.W., Song D.H., Kim Y.J., Choi Y.S., Kim C.J. (2016). Effects of fat replacement with a mixture of collagen and dietary fibre on small calibre fermented sausages. Int. J. Food Sci. Tech..

[4] Hansen P.W., Tholl I., Christensen C., Jehg H.C., Borg J., Nielsen O., Östergaard B., Nygaard J., Andersen O. (2003). Batch accuracy of on-line fat determination. Meat Sci..

[5] Prieto N., Roehe R., Lavin P., Batten G., Andres S. (2009). Application of near infrared reflectance spectroscopy to predict meat and meat products quality: A review. Meat Sci..

[6] Parliament and Council Regulation (EU) No 1169/2011 of 25 October 2011, on the Provision of Food Information to Consumers, Amending Regulations (EC) No 1924/2006 and (EC) No 1925/2006 of the European Parliament and of the Council, and Repealing Commission Directive 87/250/EEC, Council Directive 90/496/EEC, Commission Directive 1999/10/EC, Directive 2000/13/EC of the European Parliament and of the Council, Commission Directives 2002/67/EC and 2008/5/EC and Commission Regulation (EC) No 608/2004. https://eur-lex.europa.eu/legal-content/EN/TXT/HTML/?uri=CELEX:32011R1169&from=PL.

[7] Hocquette J.F., Gondret F., Baéza E., Médale F., Jurie C., Pethick D.W. (2010). Intramuscular fat content in meat-producing animals: Development, genetic and nutritional control, and identification of putative markers. Animal.

[8] Wood J.D., Enser M., Fisher A.V., Nute G.R., Sheard P.R., Richardson R.I., Hughes S.I., Whittington F.M. (2008). Fat deposition, fatty acid composition and meat quality: A review. Meat Sci..

[9] Barbin D.F., ElMasry G., Sun D.W., Allen P. (2013). Non-destructive determination of chemical composition in intact and minced pork using near-infrared hyperspectral imaging. Food Chem..

[10] ElMasry G., Nakauchi S. (2015). Noninvasive sensing of thermal treatments of Japanese seafood products using imaging spectroscopy. Int. J. Food Sci. Technol..

[11] Li X., Feng F., Gao R., Wang L., Qian Y., Li C., Zhu G. (2015). Application of near infrared reflectance (NIR) spectroscopy to identify potential PSE meat. J. Sci. Food Agric..

[12] Marcoux M., Bernier J.F., Pomar C. (2003). Estimation of Canadian and European lean yields and composition of pig carcasses by dual-energy X-ray absorptiometry. Meat Sci..

[13] Prados M., Fulladosa E., Gou P., Muñoz I., Garcia-Perez J.V., Benedito J. (2015). Non-destructive determination of fat content in green hams using ultrasound and X-rays. Meat Sci..

[14] Ghaedian R., Decker E.A., McClements D.J. (1997). Use of ultrasound to determine cod fillet composition. J. Food Sci..

[15] Nowak K.W., Markowski M., Daszkiewicz T. (2016). A modified ultrasonic method for determining the chemical composition of meat products. J. Food Eng..

[16] Vester-Christensen M., Erbou S.G.H., Hansen M.F., Olsen E.V., Christensen L.B., Hviid M., Ersbřll B.K., Larsen R. (2009). Virtual dissection of pig carcasses. Meat Sci..

[17] Adamczak L., Chmiel M., Florowski T., Pietrzak D., Witkowski M., Barczak T. (2015). A potential use of 3-D scanning to evaluate the chemical composition of pork meat. J. Food Sci..

[18] Adamczak L., Chmiel M., Florowski T., Pietrzak D., Witkowski M., Barczak T. (2018). Using density measurement on semispinalis capitis as a tool to determinate the composition of pork meat. Food Anal. Method.

[19] Brown C.J., Hiller J.C., Whatley J.A. (1951). Specific gravity as a measure of the at content of the pork carcass. J. Anim. Sci..

[20] Feder E. (1913). Eine Grundlage zur Erkennung eines übermäßigen Wasser-zusatzes zu zerkleinerten Fleischwaren. Z. Unters. Nahr. Genußmittel.

[21] Kenawi M.A., Abdelsalam R.R., El-Sherif S.A. (2009). The effect of mung bean powder, and/or low fat soy flour as meat extender on the chemical, physical, and sensory quality of buffalo meat product. Biotechnol. Anim. Husb..

[22] Stubbs B., More A. (1919). The estimation of the appropriate quantity of meat in sausages and meat pastes. Analyst.

[23] Commission Regulation (EC) No 2004/2002 of 8 November 2002, Concerning the Procedure for Meat and Fat Content Determination in Certain Pork Meat Products. https://eur-lex.europa.eu/legal-content/EN/TXT/?qid=1562245953290&uri=CELEX:32002R2004.

[24] Commission Regulation (EC) No 543/2008 of 16 June 2008, Introducing Detailed Rules for Implementing Council Regulation (EC) No 1234/2007 on Certain Marketing Standards for Poultry Meat. https://eur-lex.europa.eu/legal-content/EN/TXT/?qid=1562246376609&uri=CELEX:32008R0543.

[25] Sosa-Morales M.E., Orzuna-Espíritu R., Vélez-Ruiz J.F. (2006). Mass, thermal and quality aspects of deep-fat frying of pork meat. J. Food Eng..

[26] (2012). Anonymous: Draft Guidance Document for Competent Authorities for the Control of Compliance with EU Legislation on: Regulation (EU) No 1169/2011 of the European Parliament and of the Council of 25 October 2011, Council Directive 90/496/EEC of 24 September 1990 and Directive 2002/46/EC of the European Parliament and of the Council of 10 June 2002-with Regard to the Setting of Tolerances for Nutrient Values Declared on a Label. https://assets.publishing.service.gov.uk/government/uploads/system/uploads/attachment_data/file/212935/EU-Guidance-on-Tolerance.pdf.

[27] Association of Official Analytical Chemists (AOAC) (2000). Official Methods of Analysis of AOAC International.

